# Removing broken locking bolts

**DOI:** 10.1308/003588412X13373405386015p

**Published:** 2012-09

**Authors:** G Erturan, R Handley

**Affiliations:** Oxford University Hospitals NHS Trust,UK

Removal of broken locking bolts requires careful planning. The following tips facilitate the procedure and reduce operation time.

**Figure 1:**
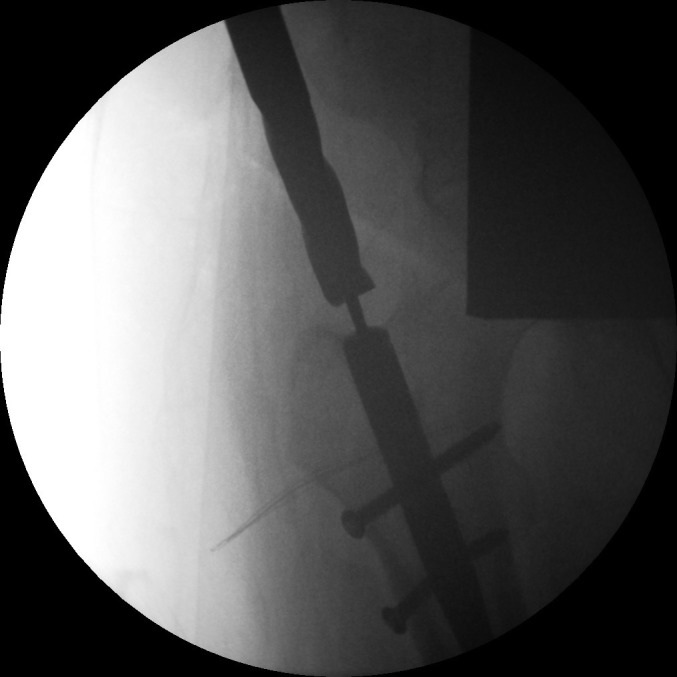
Use of a cannulated drill with guidewire to reach end cap

**Figure 2:**
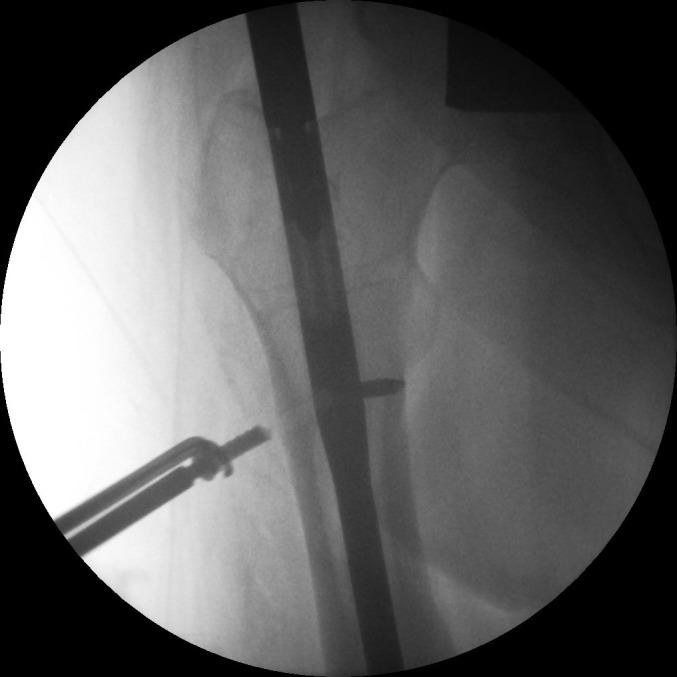
Long length screw holding clip

**Figure 3:**
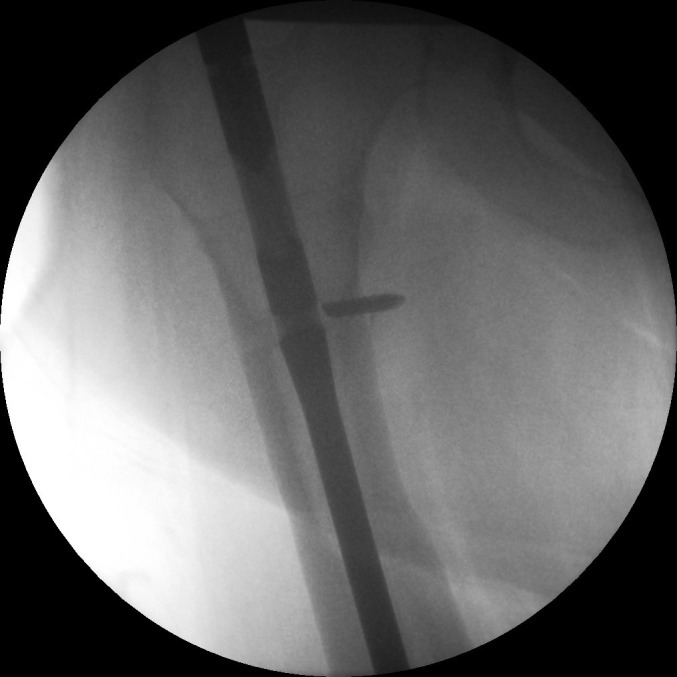
Nail retraction to aid broken screw into far cortex

**Figure 4:**
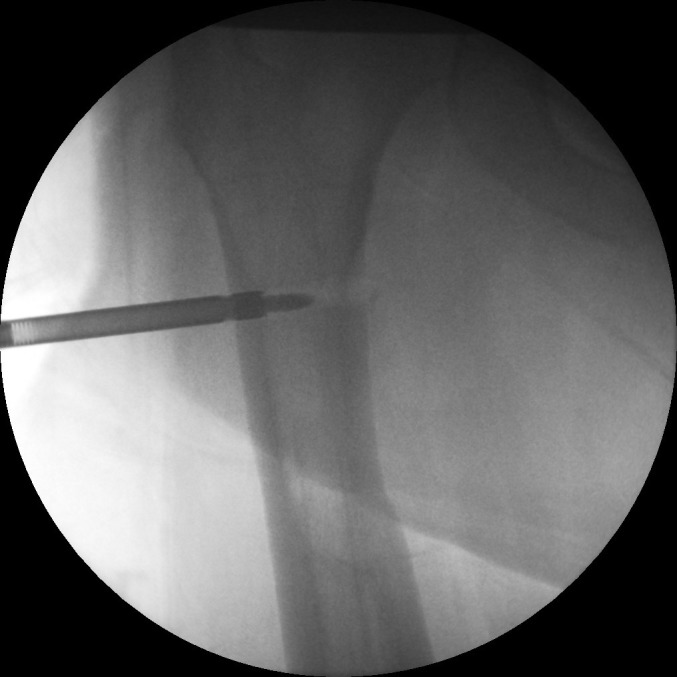
Broken screw extraction

Positioning the patient supine without traction allows optimal adduction. Early screw removal will leave the nail rotationally unstable; apply the jig beforehand. If direct palpation is not possible, access the end cap by a guided drill (Fig 1). Use screw holders (Fig 2). Retract the nail to realign the broken screw with the nail hole. Tap the fractured screw to advance it into the far cortex for purchase (Fig 3). Screws can then be removed with a broken screw set (Fig 4).

